# Cross-Disorder Genetic Analysis of Tic Disorders, Obsessive–Compulsive, and Hoarding Symptoms

**DOI:** 10.3389/fpsyt.2016.00120

**Published:** 2016-06-30

**Authors:** Nuno R. Zilhão, Dirk J. Smit, Dorret I. Boomsma, Danielle C. Cath

**Affiliations:** ^1^Department of Clinical and Health Psychology, Utrecht University, Utrecht, Netherlands; ^2^Department of Biological Psychology, Vrije Universiteit, Amsterdam, Netherlands; ^3^Neuroscience Campus Amsterdam, Vrije Universiteit, Amsterdam, Netherlands; ^4^Altrecht Academic Anxiety Center, Utrecht, Netherlands

**Keywords:** tic, hoarding, obsessive–compulsive symptoms, heritability, trivariate, twin

## Abstract

Hoarding, obsessive–compulsive disorder (OCD), and Tourette’s disorder (TD) are psychiatric disorders that share symptom overlap, which might partly be the result of shared genetic variation. Population-based twin studies have found significant genetic correlations between hoarding and OCD symptoms, with genetic correlations varying between 0.1 and 0.45. For tic disorders, studies examining these correlations are lacking. Other lines of research, including clinical samples and GWAS or CNV data to explore genetic relationships between tic disorders and OCD, have only found very modest if any shared genetic variation. Our aim was to extend current knowledge on the genetic structure underlying hoarding, OC symptoms (OCS), and lifetime tic symptoms and, in a trivariate analysis, assess the degree of common and unique genetic factors contributing to the etiology of these disorders. Data have been gathered from participants in the Netherlands Twin Register comprising a total of 5293 individuals from a sample of adult monozygotic (*n* = 2460) and dizygotic (*n* = 2833) twin pairs (mean age 33.61 years). The data on Hoarding, OCS, and tic symptoms were simultaneously analyzed in Mplus. A liability threshold model was fitted to the twin data, analyzing heritability of phenotypes and of their comorbidity. Following the criteria for a probable clinical diagnosis in all phenotypes, 6.8% of participants had a diagnosis of probable hoarding disorder (HD), 6.3% of OCS, and 12.8% of any probable lifetime tic disorder. Genetic factors explained 50.4, 70.1, and 61.1% of the phenotypic covariance between hoarding-OCS, hoarding-tics, and OCS-tics, respectively. Substantial genetic correlations were observed between hoarding and OCS (0.41), hoarding and tics (0.35), and between OCS and tics (0.37). These results support the contribution of genetic factors in the development of these disorders and their comorbidity. Furthermore, tics were mostly influenced by specific environmental factors unshared with OCS and HD.

## Introduction

Current classification systems of psychiatric disorders are primarily based on consensus statements with respect to clinical symptom diagnostics by physicians. These classification systems, i.e., the International Classification of Diseases (ICD) (1) and the Diagnostic and Statistical Manual of Mental Disorders (DSM) (2), have rendered the separate and categorical entities we know as disorders – including obsessive–compulsive disorder (OCD), Tourette’s disorder (TD), and (starting from DSM-5) hoarding disorder (HD).

More specifically, OCD, HD, and tic disorders/TD are complex neuropsychiatric disorders; all characterized by repetitive behaviors that show substantial comorbidity, i.e., co-occurring more often than expected by chance (3–6). OCD is a neurodevelopmental disorder characterized by recurrent intrusive thoughts (obsessions) and repetitive behaviors (compulsions) designed to relieve either tension or anxiety stemming from the obsessions (7, 8). HD has since long been classified as a symptom dimension of OCD, and – to a lesser extent – as a characteristic of obsessive–compulsive personality disorder (7). However, it was later suggested that (1) HD presents mostly (in up to 80% of cases) without concurrent OCD (9) and (2) the neurological mechanisms underlying hoarding might be distinct from OCD (9, 10). Therefore, it was included in DSM-5 as a distinct disorder in the category of OCD spectrum disorders and characterized by the inability to discard an excessive amount of items of no significant value, combined with excessive acquisition and clutter to such an extent that living spaces of an individual are occupied (2). Tic disorders are characterized by recurrent motor and/or vocal tics that occur in a stereotypical fashion against a background of normal motor/phonic activity, with onset in childhood and tendency to decrease in intensity and frequency during adolescence (11).

Prevalence rates for these disorders range between 0.1 and 0.8% for TD (12–19), 2 and 6% for compulsive hoarding (20, 21), and 0.5 and 2.0% for OCD (7, 22).

With respect to comorbidity rates between HD and OCD, in clinical and epidemiological studies of OCD, between 18 and 42% of patients report hoarding behaviors, depending on phenotypic definition (23–26), and reversely, in 12–20% of HD patients, OCD is reported (27–29). In TD/chronic tic disorders, OCD is very common, with estimates ranging from 28 to 49% of OCD/OC symptoms (OCS) in TD, and reversely, of 10–20% of tics in OCD (30, 31). In sum, these comorbidity estimates are well above expected comorbidity rates if the three disorders would be etiologically distinct. Finally, in tic disorders, no studies on hoarding comorbidity have been performed nor have studies been performed on tic comorbidity in HD.

Family studies and genetic epidemiological twin studies on each separate disorder have shown substantial genetic contribution to each separate phenotype, with heritability estimates from twin studies ranging between 0.30 and 0.58 (OCD) (31–35), 0.35 and 0.50 (HD) (20, 28, 33), and.25 and 0.58 (tic disorders) (36–40). A next question is whether the high proportions of co-occurrence between the three phenotypes reflect overlap in genetic or environmental contributions between OCD, HD, and tics. Multivariate twin/family studies are particularly suitable for this, making use of correlations between MZ and DZ twins on the various traits to partition the relative contribution of shared vs. unique genetic and environmental factors that influence multiple traits (41).

Despite recent advances in psychiatric genetics, twin studies specifically investigating shared genetic and environmental influences between OCS, hoarding behavior, and tics are scarce. Two studies by Iervolino et al. in a sample consisting predominantly of female twins from the TwinsUk twin registry (4459 female twins, mean age of 55.0 years) have specifically examined the genetic and environmental overlap between OCS and HD behavior (20, 33). It was found that 45% of the genetic variance was shared between HD and OCS dimensions. Furthermore, hoarding had the lowest loading on the common factor with only 55% of the total variance in OC symptom dimensions being hoarding-specific. A recent twin study of our group within the Netherlands twin Register (NTR), which overlaps with our sample, assessed the unique and shared genetic contributions for HD and OCS in a sample of 7567 twins (2270 males, 5297 females, mean age of 33.2 years) (29). The authors found significant genetic contributions to the comorbidity across both traits, although a low genetic correlation (0.10) was found. Finally, a recent population-based twin family study with data from the Swedish Twin Register (*n* = 20.821) specifically addressed the proportion of shared genetic and environmental factors underlying the liability to chronic tics, ADHD, and OCS (42). Tics were broadly defined based on the number of *total* tics (“no tic score,” “tic score = 1,” and “tic score > 1”). A substantial correlation of 0.45 between tics and OCS was found.

From another line of research, Genome-wide association study (GWAS) data from samples of TD and OCD patients were analyzed to find a genetic correlation between OCS and TD of 0.41 (43), which was relatively high in light of what has been described for other complex disorders (44). However, this correlation might have been an overestimation, as the SE of this estimate was large (SE = 0.15) and, in addition, the co-occurence between tics and OCD appeared relatively high (13% of OCD had co-occurring tics/TD, and reversely, 43% of TD had OCD) Furthermore, in this same sample, Yu et al. sought to characterize common genetic variants shared among TD and OCD. Although no specific variants were identified, the combined GWAS signals were significantly enriched for functional alleles, suggesting that there is some proportion of TD–OCD-shared genetic risk variants (45).

So far, genetic epidemiological twin family studies to estimate the shared respective unique contributions of genetic and environmental factors between tic-HD symptoms and between tic-HD–OCS are lacking, as are molecular genetic studies to estimate shared genetic contributions from SNPs across TD, OCS, and HD phenotypes.

Therefore, the main aim of this study was to extend the available data so far with respect to shared etiology between OCS and hoarding behavior (29) by expanding with the tic phenotype, in a large population-based twin sample that includes male, female, and opposite sex twin pairs using diagnostic methods that assess the full range of the symptomatology of these disorders to better address their shared underlying etiology. Specifically, we aimed at (1) replicating previous quantifications of shared and independent genetic contributions to OCS-hoarding behavior; (2) quantifying shared and independent genetic contributions to hoarding behavior and tics; (3) quantifying shared and independent genetic contributions to OCS and tics; and (4) quantifying shared and independent genetic contribution to OCS-hoarding behavior and tics.

## Materials and Methods

### Subjects

Participants included in this study are registered with the NTR. Since 1991, twins and their family members receive surveys by mail and are assessed with questionnaires about health, personality, and lifestyle (46, 47). For these analyses, we used data collected in 2008, corresponding to the survey 8 wave of collection, on obsessive–compulsive symptoms, hoarding, and tic symptoms (henceforth named as “tics”). A total of 16,930 participants from 7400 different families completed the questionnaires. Twins encompassed 8047 individuals (2511 males and 5536 females). This study has been approved by the Medical Ethical Committee of the VU Medical Center Amsterdam.

### Measurements

The assessment instruments used were the Hoarding Rating Scale-Self-Report (HRS-SR) for hoarding, the Padua Inventory Abbreviated Revised (PI-ABBR) for OCS, and an abbreviated self-report questionnaire (the Schedule for Tourette and Other Behavioral Syndromes – STOBS-ABBR) based on the Schedule for Tourette and Other Behavioral Syndromes (STOBS) for tics. The HRS-SR questionnaire consists of five items, each scoring on a 0–8 scale, that assess cluttering, difficulty in discarding items, excessive acquisition or collecting, distress derived from hoarding symptoms, and functional impairment (48). The distress item was discarded due to approval restriction on the items to be included in the larger questionnaire. The PI-ABBR questionnaire has been derived from the Padua Inventory-Revised, a 41-item self-report instrument that measures OCS on a scale from 0 to 4, and 5 subsequent subscales (washing, checking, rumination, precision, and impulses). The PI-ABBR has been abbreviated to 12 items that include 2–3 items from each of the five OCS dimensions mentioned above (49). These subscales refer to four main factors of obsessions and compulsions – “impaired control,” “fear of contamination,” “checking behavior,” and “urge/worry of losing control” (50).

The STOBS consists of a semi-structured assessment on tics and has been widely used in data collections by the Tourette Syndrome Association International Consortium for Genetics (TSAICG). It consists of 36 tic items (rated as current/lifetime, not present), generating lifetime tic information (51). For the NTR 2008 survey, the STOBS was abbreviated to a 12-item tic questionnaire on the 9 most frequent tics occurring in clinical samples (11, 52). Additionally, three items were added on age at onset of symptoms, tic severity, and whether the tic persisted for more than a year. Using the STOBS-ABBR, a diagnosis of probable chronic tic disorder was established if the person had (1) one or more chronic motor or one or more vocal tic that (2) occurred before age 21, and (3) had been present for >1 year. Probable TD diagnosis was established when two or more motor and one or more vocal tics were reported that occurred before age 21 and had lasted for >1 year, and probable transient tic disorder was established when motor and/or vocal tics had occurred before age 21 for <1 year. Participants who reported at least one tic, but without an age at onset ≤21, and/or with a tic duration of <1 year were categorized as a probable tic disorder NOS. We use the term “probable” since tic diagnoses were not confirmed by a face-to-face interview by an experienced clinician.

We fitted a liability threshold model, using, for each phenotype, a categorical variable derived from several cut points applied to the full distribution of sum scores (for OCS and HD) and defining the presence/absence of a tic disorder (for tics). The liability threshold model assumes an unobserved (and not measured) liability (or risk) to disease, normally distributed in the population (53, 54). The categories function as a (indirect) measure of this liability, representing the susceptibility to the true underlying distribution of the disease. Four categories were used for both the HRS-SR and PI-ABBR. The HRS-SR was divided into categories that more closely resemble the clinical patterns of symptomatology (no hoarding symptoms, mild symptoms, subclinical hoarding, and clinically significant hoarding or probable HD) having unequal distributions in each category (scores of 0, 1–5, 6–16, and ≥17) (20). For a probable HD diagnosis, we used the cutoff proposed by Tolin to define caseness (48). In this work, a receiver operating characteristic (ROC) analysis determined that the best threshold separating HD from non-HD cases was a sum-score over the cutoff of 17 with a sensitivity and a specificity of 0.95. The scores for PI-ABBR (0, 1–6, 7–15, and ≥16) have been previously described in the literature (49). In brief, ROC determined that the best threshold separating OCD from non-OCD cases was a sum-score over the cutoff of 16 with a sensitivity of 0.74 and a specificity of 0.72. For tics, we derived a dichotomous variable defining the presence or absence of any of the tic disorders described here above, according to a definition of “probable tic disorder,” as defined by the STOBS-ABBR. For further details on the phenotype definition for tics, please refer to (Zilhao et al., submitted^1^). Briefly, the probable tic disorder dichotomous variable consists of the most lenient definition defined for caseness, in which lifetime probable chronic tic disorder, probable TD, and probable transient tic disorder are included.

### Statistical Analysis

#### Univariate Twin Analysis

Prevalences, means, and distributions for the three phenotypes were calculated in the entire sample of 16,930 individuals. Performing these analyses on clinically defined significant symptoms has the advantage of increasing the generalizability of the results. Polychoric correlations (correlations on the liability scale) were calculated in Mplus (55) for the PI-ABBR, HRS-SR, and STOBS-ABBR, both in MZ and DZ twin pairs by sex, and in all twins for both sexes. Data from both complete and incomplete twin pairs were included in the analysis. Univariate analyses for each phenotype were performed separately using the software OpenMx (56) to estimate the relative contributions from additive genetic (A), shared environment (C), and non-shared environment (E) to each phenotype. Maximum-likelihood model fitting procedures were carried out, as is standard in structural equation modeling, in which the phenotype was a function of the A, C, and E factors and polychoric correlations, according to the liability threshold model described above. We investigated the potential influence of twin-specific and gender-specific (sex differences) environment by constraining correlations across zygosity groups to be equal, for all three phenotypes. The effect of covariates (age and sex) on the thresholds was univariately assessed for each phenotype.

#### Multivariate Twin Analyses

Using the Mplus software, we then fitted a trivariate genetic model to the data with the weighted least square mean and variance adjusted estimation option (WLSMV) (55), using the described liability threshold models. Covariances between the three phenotypes were partitioned into the relative contributions of shared additive genetic (A), common environmental (C), and non-shared environmental (E) influences to the etiology of the three phenotypes. The influence of common environmental factors and of genetic dominance were tested by comparing a nested AE model with either the ACE or the ADE model using the Chi-square difference test.

Lastly, we performed a single factor analysis on the covariance matrices partitioned between the phenotypes. This analysis gives a representation in terms of the components shared by the three phenotypes.

## Results

### Descriptives

#### Means and Distributions

The mean age of the entire sample was 33.61 years (SD = 14.56); for males the mean age was 33.11 years (SD = 14.66) and for females 33.84 years (SD = 14.51). The mean average score for HRS-SF was 5.74 (SD = 5.6) and for the PI-ABBR was 6.89 (SD = 5.2). Males had on average higher scores than females on both the HRS-SF and the PI-ABBR. Also for tics, the prevalence rates were higher in males (13.0%) than in females (12.6%). Table 1 summarizes the demographics in males and females for the PI-ABBR, HRS-SF, and STOBS-ABBR.

**Table 1:** **Sample demographics for the data included in the analysis**.

	MZ twins		DZ twins	
	Male	Female	Male	Female
Mean age	35.09 (15.27)	35.63 (15.20)	31.57 (13.98)	31.88 (13.45)
Mean HRS	5.85	5.5	6.08	5.79
Mean PADUA	6.99	6.7	7.04	6.99
Tics (prevalence)	192	188	175	162

#### Prevalence and Phenotype “Overlap”

Table 2 shows prevalence rates for the three phenotypes for MZ and DZ twins, as estimated according to the diagnostic criteria. Of the entire sample, 5.0% had clinically significant HD, 6.0% had clinically significant OCS, and 13.5% had any probable tic disorder according to the STOBS-ABBR. The threshold used to determine caseness in a probable HD disorder diagnosis rendered population prevalence rates that closely resemble previous estimates for clinical HD (20, 21). Furthermore, among individuals with OCS, 18.0% had co-occurring HD and 12.1% had tics; among individuals with HD, 15.0% had OCS and 8.72% had tics; among individuals with tics, 27.1% had OCS and 23.3% had HD. Lastly, in the entire sample, 0.31% (*n* = 25) of individuals had the co-occurrence of all three disorders.

**Table 2:** **Prevalence rates for HD (HRS-SR), OCS (PI-R-ABBR), and tics (YGTSS) for the total sample included in the analysis**.

	Category	MZ (*n* = 3990) *N* (%)	DZ (*n* = 4057) *N* (%)
HD (*n* = 5221) symptom scores	0	673 (22.8)	435 (19.1)
	1–5	1059 (36)	826 (36.2)
	6–16	1079 (36.6)	880 (38.6)
	>16	137 (4.6)	132 (5.7)
OCS (*n* = 5167) symptom scores	0	190 (6.5)	140 (6.3)
	1–4	1447 (49.6)	1077 (48.0)
	5–15	1107 (37.9)	898 (39.9)
	>15	175 (6.0)	133 (5.9)
Probable tic disorder (*n* = 5297) affected/non-affected	TD Chr motor tic Chr vocal tic Transient tic disorder Tic disorder NOS	15 (0.5) 39 (1.3) 17 (0.6) 159 (5.4) 150 (5.0)	14 (0.6) 35 (1.5) 16 (0.7) 138 (5.9) 134 (5.7)

### Univariate Results

#### Twin Correlations

Table 3 shows the polychoric correlations as calculated on the observed data for the five zygosity groups, on the HRS-SR, PI-ABBR, and the STOBS-ABBR. Overall, when comparing MZ and DZ pairs on the three phenotypes, an average twofold increase for MZ twins when compared to DZ twins is observed. The greater similarity for MZ twins is an indication of a genetic basis influencing the phenotypes. Also, the moderate MZ correlations suggest the influence of non-shared environmental factors for all three phenotypes.

**Table 3:** **Polychoric twin correlations for observed data for HD, OCS, and tics**.

	MZ	MZM	MZF	DZ	DZM	DZF	DOS
HD (HRS-SR)	0.336	0.379	0.325	0.177	0.247	0.151	0.048
OCS (PI-R-ABBR)	0.384	0.379	0.386	0.177	0.197	0.139	0.214
Tics (STOBS)	0.37	0.242	0.414	0.19	0.238	0.172	0.114

Specific gender/twin environments were tested univariately for each phenotype. As expected from the twin correlations across all zygosities, the fit statistics results show that correlations could be equated across twins and sex, with no twin-specific or sex-specific environments observed (Table 4).

**Table 4:** **Model fit indices for the univariate models, examining the role of sex and zygosity, of each phenotype separately**.

Model	NP	−2LL	Versus model	χ^2^	df	*p*
1. Hoarding, saturated	10	–	–	–	–	–
2. Hoarding, equal sex, and zygosities	7	3250.51	Hoarding, saturated	3.83	3	0.28
3. OCS, saturated	10	–	–	–	–	–
4. OCS, equal sex, and zygosities	7	840.18	OCS, saturated	1.07	3	0.78
5. Tics, saturated	10	–	–	–	–	–
6. Tics, equal sex, and zygosities	7	16,091.12	Tics, saturated	5.45	3	0.14

*NP, number of parameters; −2LL, −2 × log-likelihood; df, degrees of freedom for χ^2^ test*.

#### Heritabilities and Fit Statistics

The total heritability estimates were 0.33 (SE = 0.05, *p* < 0.001) for clinically significant HD, 0.38 (SE = 0.05, *p* < 0.001) for OCS, and 0.37 for any tic disorder (SE = 0.05, *p* < 0.001) (off-diagonal in Table 5). For non-shared environment, the estimates were 0.67 (SE = 0.05, *p* < 0.001) for clinically significant HD, 0.62 for OCS (SE = 0.05, *p* < 0.001), and 0.63 (SE = 0.05, *p* < 0.001) for tics. No evidence was found for an effect of common environment.

**Table 5:** **Relative contributions of additive genetic and non-shared environmental influences on the trait variance (diagonal) and covariance cross-trait (off-diagonal) for HD (HRS-SR), OCS (PI-R-ABBR), and tics (YGTSS)**.

	Phenotypic correlation		CTCT (MZ below, DZ above diagonal)			Additive genetic effects (A)			Non-shared environmental effects (E)		
	HD	OCS	HD	OCS	Tics	HD	OCS	Tics	HD	OCS	Tics
HD	–		–	0.07	0.05	0.326	–	–	0.674	–	–
OCS	0.3	–	0.14	–	0.02	0.504	0.375	–	0.496	0.625	–
Tics	0.15	0.25	0.12	0.16	–	0.701	0.611	0.367	0.299	0.389	0.633

*CTCT, cross-twin-cross-trait correlations*.

### Cross-Disorder Correlations

Examining the cross-disorder correlations (cross-twin cross-trait) again suggests that the genetic factors are involved in the correlations between traits (Table 5). The MZ cross-twin cross-trait correlations were 0.14 (HD vs. OCS), 0.12 (HD vs. tics), and 0.16 (OCS vs. tics), while the DZ correlations were 0.07 (HD vs. OCS), 0.05 (HD vs. tics), and 0.02 (OCS vs. tics). The within-person cross-trait correlations (phenotypic correlation) were 0.30 (HD vs. OCS), 0.15 (HD vs. tics), and 0.25 (OCS vs. tics) (Table 5).

**Figure 1:**
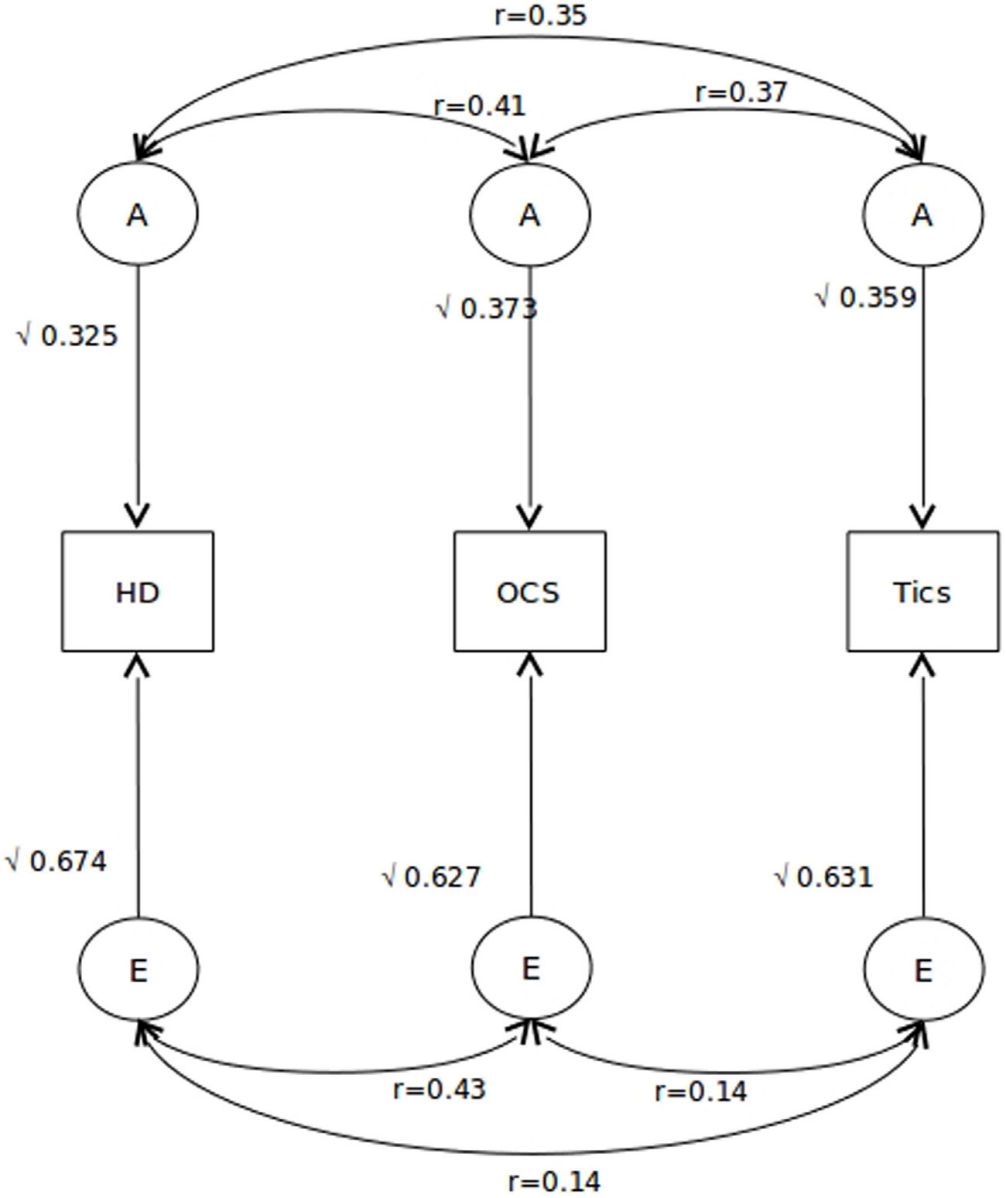
**Path diagram for the best-fitting model**. Squaring these paths gives the proportion of variance accounted by each of the A and E components. Also indicated are the correlations among each A and E component for each of the three phenotypes. A indicates additive genetic factors and E indicates non-shared environmental factors.

A trivariate ACE model was fitted to the data in order to examine the relative contributions from shared genetic and environmental contributions to the covariance among the traits. Again, as suggested by patterns of twin correlations, no evidence for common environment was found, and the C parameter could be dropped when compared to the more parsimonious AE model [AE vs. ACE model: χ^2^ (6) = 0.876, *p* = 0.99 and AE vs. ADE model: χ^2^ (6) = 2.994, *p* = 0.81]. Hence, the best-fitting model to the data was one in which the covariation between the three phenotypes can be explained by a set of common A and E factors. Table 5 and Figure 1 show the estimates of the relative contributions of genes and non-shared environment factors, calculated from the best-fitting model. The total variance for each variable was constrained to 1, in order to estimate the proportion of individual liability due to shared vs. common genetic/environmental factors. Bivariate heritability results (Table 5) show that 50% of the covariance between HD and OCS, 70% of the covariance between HD and tics, and 61% of the covariance between OCS and tics are due to genetic factors. The remaining variance is accounted for by non-shared environmental factors. Furthermore, the genetic correlations were 0.41 (HD vs. OCS), 0.35 (HD vs. tics), and 0.37 (OCS vs. tics). Figure 1 depicts the path diagram in terms of correlated A and E factors.

**Figure 2:**
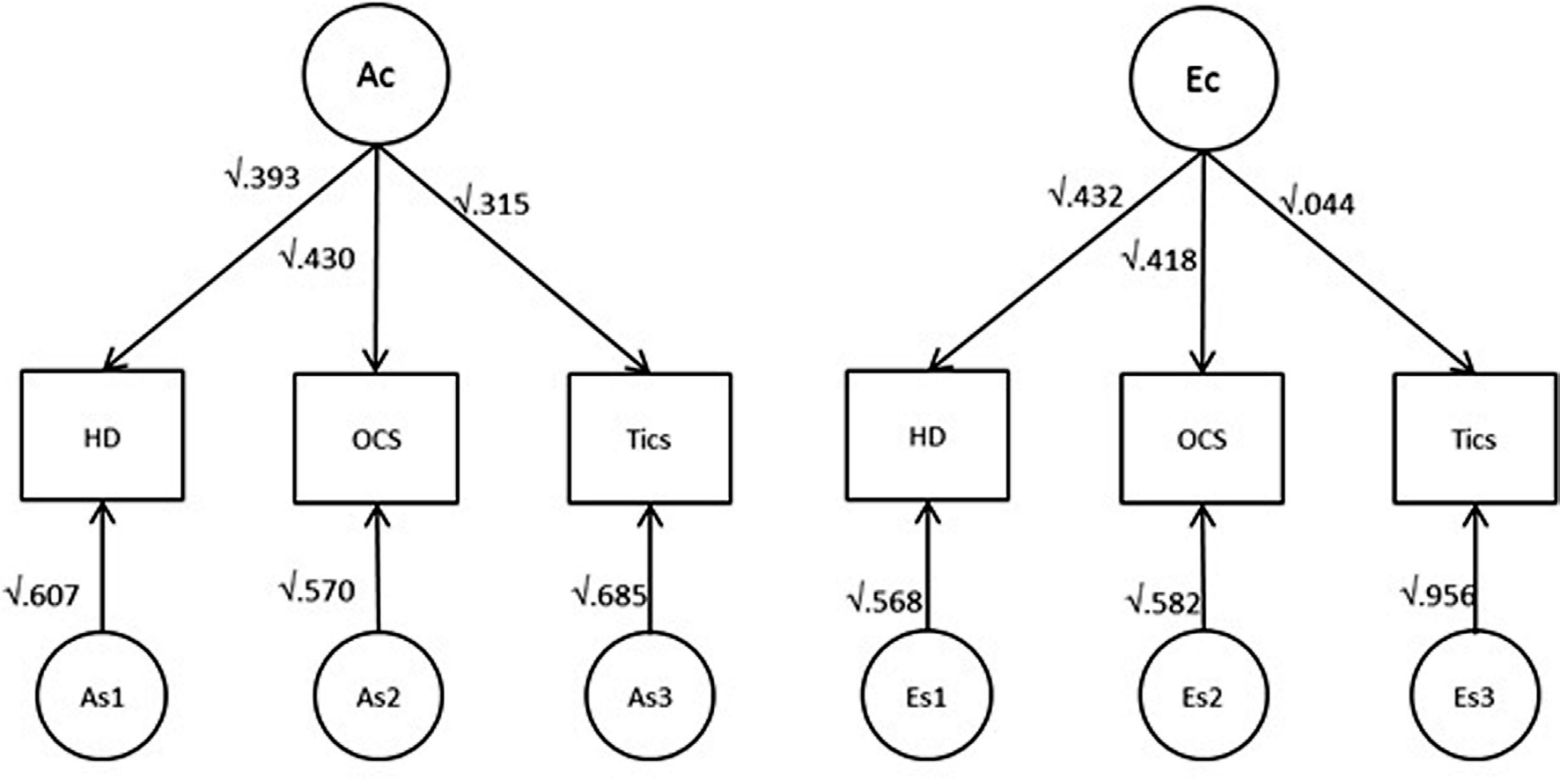
**Single factor representation for the each of the A and E component for the best-fitting model**. Numbers indicate the proportion (for both A and E components) shared by the three phenotypes. Ac indicates common additive genetic factors and Ec indicates common non-shared environmental factors.

Lastly, single factor analysis for the A and E component revealed the degree of genetic and environmental overlap shared by the three phenotypes (Figure 2). As shown, between 31.5 and 43% of the total genetic variance of each phenotype is due to genetic factors shared among all three phenotypes. Specific genetic variance unshared with other phenotypes was 60.7% (HD), 57.0% (OCS), and 68.5% (tics). Furthermore, 43.2 and 41.8% of the total environmental variance is due to unique environmental factors shared between HD and OCS, respectively, whereas for tics, this amounts only to 4.4% of the total environmental variance – in other words, tics had the lowest loading on the common factor and were mostly influenced by tic-specific environmental effects (Figure 2).

## Discussion

In this study, we sought to examine the extent to which shared genetic and environmental factors contribute to clinically significant OCS, HD, and tic symptomatology. We had at our disposal the largest twin pair sample available to date in which these three phenotypes were measured at the same wave of data collection. The present results extend previous work in the same NTR sample on shared genetic contributions to OCS and HD (29).

Our univariate prevalence rates for clinical significant HD symptoms and OCS are in the expected range when compared to the literature (49, 57). For tics, we note that our somewhat higher prevalence rates than described in the literature might be due to the fact that they reflect lifetime tic disorders, and therefore a somewhat lenient definition for caseness, reflecting our approach to generate optimal results with respect to phenotypic validity, in light of the self-report measures used in the NTR.

Our comorbidity prevalence rates (8.0% of OCS patients reported co-occurring HD, and reversely, 15.0% of HD patients reported co-occurring OCS; 12.1% of OCS patients reported co-occurring tics, and reversely, 27.1% of TD/chronic tic disorders reported co-occurring OCS) are within the expected range when compared with the epidemiological literature (23–31). For HD/tics, to the best of our knowledge, we report here the first comorbidity prevalence rate estimate – 8.72% of HD individuals having co-occurring tics and 23.3% of tic individuals having HD.

### The Univariate Model Fitting Results

Previous results with data from the NTR, using by and large the same sample, have yielded heritabilities of 0.40–0.50 for OCS (49, 58), 0.36 for HD (29), and 0.30 for tics (see footnote text 1). Other previous twin/family studies have rendered comparable estimates (0.26–0.55 for OCS, 0.35–0.50 for hoarding, and 0.28–0.56 for tics) (42, 59, 60). We found no evidence for sex differences in twin correlations for any of the phenotypes. Similar findings have been reported for OCS (32, 33, 61), whereas for HD results have been mixed (20, 28); for tics, to the best of our knowledge, the issue of sex differences in twin correlations has not yet been addressed. Our results here show that the genetic contributions to these phenotypes are consistent across both sexes.

### Bivariate Analyses

Second, our results provide evidence for shared genetic variation between the phenotypes. The phenotypic correlation between OCS and HD was of 0.30. As expected, we observed a higher phenotypic correlation between OCS and tics (0.25) than between HD and tics (0.15). The genotypic correlations also mirrored this – there was higher shared genetic variance between OCS and HD (0.41) than both OCS and HD with tics (0.37 and 0.35, respectively). Interestingly, a relatively high proportion of the phenotypic correlations were attributable to genetic factors. In other words, although the genetic overlap (expression of same genes) between tics and both OCS and HD is moderate, a substantial proportion of the phenotypic correlation is mediated by their shared genetic variance (61 and 70%, respectively).

Importantly, Iervolino et al. recently reported a genetic correlation between OCS and HD of 0.45, combined with their data suggesting that HD was mostly influenced by specific genetic effects (54.5% specific) (33). The authors argued that this supports the notion of these disorders constituting two etiologically distinct, although related, entities (20, 33). Furthermore, Mathews et al. reported a substantially lower genetic correlation of 0.10 (29). Our current findings of all cross-twin cross-trait genetic correlations being below 0.2 and the within-person cross-trait correlations being all below 0.35 are mostly in line with those in the study by Iervolino et al. (33). Iervolino et al. argue that the magnitude of these genetic correlations is lower than the shared genetic variance of 0.55 between OCD and other internalizing disorders. i.e., panic disorder, generalized anxiety, phobias, and PTSD (33, 62). They reason that a genetic overlap just under 0.50 argues in favor of HD being a separate, but related entity, as it is currently defined in DSM-5. Our data on the relationship between HD and OCS are in support of this view.

Our estimates of genetic correlations between OCS and tics (0.37) are somewhat lower than the genetic correlations (0.45) as found by Pinto et al. (42). The differences in estimates might be explained by the different phenotypic tic definitions requiring an age of onset before 21 resulting in a prevalence of 13.5%, whereas their multinomial definition of lifetime tics into categories “no tic,” “one tic,” and “two or more tics” resulted in prevalences of 16% at the first and 6% at the second threshold. Furthermore, our results are not fully in line with tic/OCS-enriched clinical family studies reporting very high genetic correlations between TD and OCS (genetic correlation = 0.92), although the SEs in this study were high (SE = 0.42) (39).

With respect to the shared genetic and environmental contributions to HD and tics, to our knowledge, this is the first twin-family study partitioning the covariance between tics and HD in its relative genetic and environmental components. Our moderate correlation estimate (0.35) supports the argument of viewing TD as distinct from HD.

Third, the common factor model further supports the view of shared genetic etiology between the three phenotypes. Neuroimaging studies have reported structural and functional dysfunctions in the cortico–striato–thalamo–cortical (CSTC) circuitries across all three disorders that have negative implications for motor response inhibition and interference control in these disorders, which might underlie the phenotypic behaviors of all these three disorders (63–65). Our results raise the interesting possibility that a common genetic architecture defines underlying CSTC dysfunctions across the three disorders. Follow-up genome-wide studies may investigate whether specific genetic variants involved in all three disorders are differentially expressed in these brain areas as a result of non-shared environmental influences. In support of this, interestingly, OCS and HD showed low environmental correlations with tics, suggesting that tic disorders have specific environmental contributors invoking tic symptoms. In other words, non-familial (unique) environmental experiences may determine the development of tics, separately from the broader obsessive–compulsive-related disorders, as currently defined in DSM-5 (2).

Finally, our results are relevant for the field of molecular genetics. The lack of power to detect specific genetic risk variants is a recurrent issue in genome-wide studies. One way to overcome this limitation is to combine related phenotypes therefore increasing sample sizes, with consequent power gains. A crucial point here is the balance between power gains from increased sample sizes and power losses from increased heterogeneity (44, 66). Our results suggest that although these disorders share substantial genetic overlap, a substantial proportion of the genetic risk variance contributing to the liability to each disorder is independent from each other, and care should be taken when combining the phenotypes as studied in this paper.

### Limitations

These results should be considered in the light of some limitations, mainly considering the phenotypes. Because this is a population-based study, the data collected are based on self-report measures, rather than on clinician-administered structural interviews. The cutoffs have been empirically derived, and are therefore somewhat arbitrary. The cutoffs to determine symptom thresholds (in the case of OCS and HD), by considering the entire range of age available, may have rendered different prevalence estimates, which might have affected estimations of genetic and environmental effects. However, we note that although these threshold cut-offs do not represent definite clinical diagnoses, they do correspond to clinical significant symptoms. Moreover, investigation of dimensions rather than true/false categorical diagnosis is consistent with the ideas forwarded in the NIMH Research Domain Criteria (RDoC) (67).

To conclude, OCS, HD, and tics share etiologic variance that can be explained by substantial genetic correlations. Tics are mostly influenced by specific environmental effects unshared with neither OCS or HD, suggesting that specific environmental stressors might cause the development of tics separate from OCS and HD. Our results are in line with the literature supporting the current definition in DSM-5 of separating these disorders into different, although related, entities.

## Author Contributions

All authors listed, have made substantial, direct and intellectual contribution to the work, and approved it for publication.

## Conflict of Interest Statement

The authors declare that the research was conducted in the absence of any commercial or financial relationships that could be construed as a potential conflict of interest.

## Footnotes

^1^Zilhao NR, Olthof MC, Smit DJA, Cath DC, Mathews CA, Delucchi K, et al. Heritability of Tic Disorders: a Twin-Family Study (submitted).
